# Correction to [Silicone dressing combined with topical oxygen therapy alleviates incontinence‐associated dermatitis via NF‐κB p65/STAT1 signaling pathway]

**DOI:** 10.1111/srt.70058

**Published:** 2024-09-19

[Guiyuan Chen, Yingxun Chen, Yan Zhang, Shufeng Zheng, Louying Zhu, Mingxing Ding. *Skin Res Technol*. 2024; 30(8):e13888.]


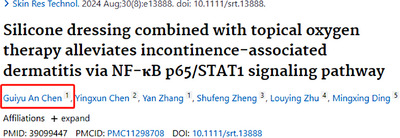


[In the author list, the name of the first author “Guiyu An Chen” is incorrect. There is no spacing between Guiyu and An. This should have read “Guiyuan Chen”.]

We apologize for this error.

